# Supplementary material for: Younger and rural children are more likely to be hospitalized for SARS-CoV-2 infections
Source: PLoS One. 2024 Oct 2;19(10):e0308221. doi: 10.1371/journal.pone.0308221 (PMC11446435; doi:10.1371/journal.pone.0308221)
Supplement: S1 Table — For this reason, we wanted to evaluate the demographic factors associated with these patients. (DOCX) [file pone.0308221.s001.docx]

**Supplemental Table 1. Demographic factors associated with the transition zones left out of the study.**

| **Demographic Factor** (Whole study) | Transition #1 (N=18) | **Transition #2 (N=58)** | **Transition #3 (N=10)** |
| --- | --- | --- | --- |
| **Age in years** |  |  |  |
| Mean ±Standard Deviation | 4.94 ±6.07 | 5.78 ±5.78 | 5.00 ±5.56 |
| Median (1^st^ – 3^rd^Quartiles) | 2.5 (0 – 11) | 3.5 (0 – 10) | 3 (0 – 11) |
| **CDC Age Group, % (N)** |  |  |  |
| Under 1 year of age | 44% (8) | 22% (13) | 40% (4) |
| 1–4 years of age | 17% (3) | 33% (19) | 10% (1) |
| 5–14 years of age | 28% (5) | 34% (20) | 50% (5) |
| 15–18 years of age | 11% (2) | 10% (6) | 0% (0) |
| **Sex, % (N)** |  |  |  |
| Female | 50% (9) | 47% (27) | 60% (6) |
| Male | 50% (9) | 53% (31) | 40% (4) |
| **Race, % (N)** |  |  |  |
| Hispanic | 11% (2) | 24% (14) | 30% (3) |
| Non-Hispanic Black | 28% (5) | 16% (9) | 20% (2) |
| Non-Hispanic White | 56% (10) | 50% (29) | 40% (4) |
| Other | 6% (1) | 10% (6) | 10% (1) |
| **Urban^1^, % (N)** |  |  |  |
| Urban | 89% (16) | 86% (50) | 90% (9) |
| Non-Urban | 11% (2) | 14% (8) | 10% (1) |
| Unknown | 0% (0) | 0% (0) | 0% (0) |
| **Insurance Payor Category, % (N)** |  |  |  |
| Commercial | 11% (2) | 16% (9) | 20% (2) |
| Medicaid | 89% (16) | 78% (45) | 80% (8) |
| Other/Self/Unknown | 0%(0) | 7% (4) | 0% |
