# Supplementary material for: Younger and rural children are more likely to be hospitalized for SARS-CoV-2 infections
Source: PLoS One. 2024 Oct 2;19(10):e0308221. doi: 10.1371/journal.pone.0308221 (PMC11446435; doi:10.1371/journal.pone.0308221)
Supplement: S2 Table — For this reason, we wanted to evaluate the disease characteristics associated with these patients. (DOCX) [file pone.0308221.s002.docx]

**Supplemental Table 2. Disease Characteristics associated with the transition zones left out of the study.**

| **Disease Characteristic** (Whole study) | Transition #1 (N=18) | **Transition #2 (N=58)** | **Transition #3 (N=10)** |
| --- | --- | --- | --- |
| **Admitted, % (N)** |  |  |  |
| No | 89% (16) | 84% (49) | 90% (9) |
| Yes | 11% (2) | 16% (9) | 10% (1) |
| *Observation Unit* | *6% (1)* | *5% (3)* | *0% (0)* |
| *Inpatient* | *6% (1)* | *9% (5)* | *10% (1)* |
| *Both* | *0% (0)* | *2% (1)* | *0% (0)* |
| **Principal Diagnosis, % (N)** |  |  |  |
| COVID-19 Only | 94% (17) | 100% (58) | 100% (10) |
| More Serious | 6% (1) | 0% (0) | 0% (0) |
| *MIS-C* | *0% (0)* | *0% (0)* | *0% (0)* |
| *Systemic C-T Involvement* | *0% (0)* | *0% (0)* | *0%(0)* |
| *COVID-19 Pneumonia* | *6% (1)* | *0% (0)* | *0% (0)* |
| **Any Complex C.C.^1^, % (N)** |  |  |  |
| No | 94% (17) | 86% (50) | 90% (9) |
| Yes | 6% (1) | 14% (8) | 10% (1) |
| **ICU Use?, % (N)** |  |  |  |
| No | 100% (18) | 95% (55) | 100% (10) |
| Yes | 0% (0) | 5% (3) | 0% (0) |
| **Length of Stay, days** |  |  |  |
| Mean ±Standard Deviation | 0.33 ±0.84 | 0.57 ±1.57 | 0.30 ±0.48 |
| Median (1^st^ – 3^rd^ Quartiles) | 0 (0 – 0) | 0 (0 – 0) | 0 (0 – 1) |
| **Return visit ≤7 days?, % (N)** |  |  |  |
| No | 89% (16) | 90% (52) | 90% (9) |
| Yes | 11% (2) | 10% (6) | 10% (1) |
| **Return visit ≤30 days?, % (N)** |  |  |  |
| No | 89% (16) | 90% (52) | 80% (8) |
| Yes | 11% (2) | 10% (6) | 20% (2) |
| **Second Infection?, % (N)** |  |  |  |
| No | 100% (18) | 97% (56) | 90% (9) |
| Yes | 0% (0) | 3% (2) | 10% (1) |
