# Supplementary material for: Younger and rural children are more likely to be hospitalized for SARS-CoV-2 infections
Source: PLoS One. 2024 Oct 2;19(10):e0308221. doi: 10.1371/journal.pone.0308221 (PMC11446435; doi:10.1371/journal.pone.0308221)
Supplement: Attachment — Submitted filename: Response to Reviewers.docx [file pone.0308221.s003.docx]

**Editorial Review.**

1. Please ensure that your manuscript meets PLOS ONE's style requirements, including those for file naming. The PLOS ONE style templates can be found at
   [https://journals.plos.org/plosone/s/file?id=wjVg/PLOSOne_formatting_sample_main_body.pdf](https://urldefense.com/v3/__https:/journals.plos.org/plosone/s/file?id=wjVg*PLOSOne_formatting_sample_main_body.pdf__;Lw!!LFqOYw!utnqI6L7BPzCC5UNUQ2PtMmbI6uivF7o-P7y5OT_ugqQCccMgOxrIIgmINuVpMm-aUH10-40SGwf0ygBGw0_Dg$) and 
   [https://journals.plos.org/plosone/s/file?id=ba62/PLOSOne_formatting_sample_title_authors_affiliations.pdf](https://urldefense.com/v3/__https:/journals.plos.org/plosone/s/file?id=ba62*PLOSOne_formatting_sample_title_authors_affiliations.pdf__;Lw!!LFqOYw!utnqI6L7BPzCC5UNUQ2PtMmbI6uivF7o-P7y5OT_ugqQCccMgOxrIIgmINuVpMm-aUH10-40SGwf0yjnQQ65FA$)

**This has been completed.**

2. We note that the grant information you provided in the ‘Funding Information’ and ‘Financial Disclosure’ sections do not match.  When you resubmit, please ensure that you provide the correct grant numbers for the awards you received for your study in the ‘Funding Information’ section.

**This has been completed.**

3. Thank you for stating the following financial disclosure: "Center for Translational Pediatric Research (NIH/NIGMS P20GM121293)
   The Translational Research Institute (NIH/NCATS UL1TR003107)"
   Please state what role the funders took in the study.  If the funders had no role, please state: ""The funders had no role in study design, data collection and analysis, decision to publish, or preparation of the manuscript."" 
   If this statement is not correct you must amend it as needed. 
   Please include this amended Role of Funder statement in your cover letter; we will change the online submission form on your behalf.

**This has been completed and can be found in our cover letter and on the title page.**

4. Thank you for stating the following in the Acknowledgments Section of your manuscript: "This work was supported by the Center for Translational Pediatric Research (NIH/NIGMSP20GM121293) at Arkansas Children’s Hospital and by the Translational Research Institute (NIH/NCATS UL1TR003107) at the University of Arkansas for Medical Sciences"
   We note that you have provided funding information that is not currently declared in your Funding Statement. However, funding information should not appear in the Acknowledgments section or other areas of your manuscript. We will only publish funding information present in the Funding Statement section of the online submission form. 
   Please remove any funding-related text from the manuscript and let us know how you would like to update your Funding Statement. Currently, your Funding Statement reads as follows: 
    "Center for Translational Pediatric Research (NIH/NIGMS P20GM121293)
   The Translational Research Institute (NIH/NCATS UL1TR003107)" Please include your amended statements within your cover letter; we will change the online submission form on your behalf.

**This has been completed. In fact, the acknowledgements section has been removed completely.**

5. Thank you for stating the following in the Competing Interests section:
   " Dr. Kennedy has been a consultant for Genentech for asthma. Funding organizations were not involved in the design or conduct of the study, nor were they engaged in the collection, analysis, or interpretation of the data, nor were they involved in the preparation, editing, or censuring of the manuscript." Please confirm that this does not alter your adherence to all PLOS ONE policies on sharing data and materials, by including the following statement: ""This does not alter our adherence to  PLOS ONE policies on sharing data and materials.” (as detailed online in our guide for authors [http://journals.plos.org/plosone/s/competing-interests).](https://urldefense.com/v3/__http:/journals.plos.org/plosone/s/competing-interests).__;!!LFqOYw!utnqI6L7BPzCC5UNUQ2PtMmbI6uivF7o-P7y5OT_ugqQCccMgOxrIIgmINuVpMm-aUH10-40SGwf0yhsdHF8cA$)  If there are restrictions on sharing of data and/or materials, please state these. Please note that we cannot proceed with consideration of your article until this information has been declared.  Please include your updated Competing Interests statement in your cover letter; we will change the online submission form on your behalf.

**This has been completed and is included on the title page and in the cover letter as requested.**

6. We note that you have indicated that there are restrictions to data sharing for this study. For studies involving human research participant data or other sensitive data, we encourage authors to share de-identified or anonymized data. However, when data cannot be publicly shared for ethical reasons, we allow authors to make their data sets available upon request. For information on unacceptable data access restrictions, please see [http://journals.plos.org/plosone/s/data-availability#loc-unacceptable-data-access-restrictions.](https://urldefense.com/v3/__http:/journals.plos.org/plosone/s/data-availability*loc-unacceptable-data-access-restrictions.__;Iw!!LFqOYw!utnqI6L7BPzCC5UNUQ2PtMmbI6uivF7o-P7y5OT_ugqQCccMgOxrIIgmINuVpMm-aUH10-40SGwf0yipQa13cg$)

**This has been completed.**

Before we proceed with your manuscript, please address the following prompts:

a) If there are ethical or legal restrictions on sharing a de-identified data set, please explain them in detail (e.g., data contain potentially identifying or sensitive patient information, data are owned by a third-party organization, etc.) and who has imposed them (e.g., a Research Ethics Committee or Institutional Review Board, etc.). Please also provide contact information for a data access committee, ethics committee, or other institutional body to which data requests may be sent.

**This has been completed.**

**Data Availability Statement:** Because of the confidential nature of some of the data and our institutional review board (IRB) restrictions, data will only be provided upon request and with an approved IRB protocol. Data requests can be sent to Dr. Rebecca Cantu (corresponding author).

b) If there are no restrictions, please upload the minimal anonymized data set necessary to replicate your study findings to a stable, public repository and provide us with the relevant URLs, DOIs, or accession numbers. Please see [http://www.bmj.com/content/340/bmj.c181.long](https://urldefense.com/v3/__http:/www.bmj.com/content/340/bmj.c181.long__;!!LFqOYw!utnqI6L7BPzCC5UNUQ2PtMmbI6uivF7o-P7y5OT_ugqQCccMgOxrIIgmINuVpMm-aUH10-40SGwf0yhxetHmoA$) for guidelines on how to de-identify and prepare clinical data for publication. For a list of recommended repositories, please see [https://journals.plos.org/plosone/s/recommended-repositories](https://urldefense.com/v3/__https:/journals.plos.org/plosone/s/recommended-repositories__;!!LFqOYw!utnqI6L7BPzCC5UNUQ2PtMmbI6uivF7o-P7y5OT_ugqQCccMgOxrIIgmINuVpMm-aUH10-40SGwf0ygmVDExOg$). You also have the option of uploading the data as Supporting Information files, but we would recommend depositing data directly to a data repository if possible.

Please update your Data Availability statement in the submission form accordingly.

**This has been completed. See above.**

7. Please amend the manuscript submission data (via Edit Submission) to include author Suzanne House.

**This has been completed.**

8. We notice that your supplementary tables are included in the manuscript file. Please remove them and upload them with the file type 'Supporting Information'. Please ensure that each Supporting Information file has a legend listed in the manuscript after the references list.

**This has been completed.**

9. Please review your reference list to ensure that it is complete and correct. If you have cited papers that have been retracted, please include the rationale for doing so in the manuscript text, or remove these references and replace them with relevant current references. Any changes to the reference list should be mentioned in the rebuttal letter that accompanies your revised manuscript. If you need to cite a retracted article, indicate the article’s retracted status in the References list and also include a citation and full reference for the retraction notice.

**This has been completed. Upon my review, I do not see any references that have been retracted. We have changed reference 27 per the reviewer’s concerns (see below). We have also altered reference 15 as the full author was not evident. Finally, we added references 14 and 17 because of the reviewer’s concerns about 30-day infection intervals for secondary infections.**

**Reviewer #1.**

This study performed a comprehensive retrospective review at a single center and using the Pediatric Hospital Information System (PHIS) on pediatric cases of SARS-CoV-2 infection leading to hospitalization. This study delineated demographic information and odds ratio for hospitalization, with different subgroup analyses (such as age group, race and gender). Overall, these comprehensive review and data analysis were performed well. There are some minor suggestions that can further improve the manuscript prior to publication.

1. In line 41, the manuscript stated the children that presented to ACH between March 1, 2020, and April 28, 2022 were included. However, in line 71, the start date of pre-Delta period is stated as May 27, 2020. Furthermore, in line 109, the manuscript stated that cases identified were between May 28, 2020 and April 28, 2022. The authors should explain the reasons of differences in the start dates of case identification.

**We appreciate the reviewer’s concerns. After review of our data, we realized our mistake. The start of the data collection for the PHIS dataset was May 27, 2020. We have made changes to the manuscript to reflect this.**

2. Section ‘Data preprocessing’, a cut-off of 30 days was used to differentiate initial and second infection. It is necessary to explain, preferably with reference(s), to support the use of such a cut-off.

**We appreciate the reviewer's comments. We utilized the 30-day cut-off for this manuscript because this is the time period that the Centers for Medicare and Medicaid Services considers a readmission for hospital billing purposes. We also used this cut-off in our previous paper (now cited at this point). We recognize that the CDC defines reinfection after 90 days of the first exposure. However, many of these patients were admitted not on their first positive but on their second. CDC also says that the evolving variants increase your risk of reinfection, which can happen as early as “several weeks after a previous infection, although this is rare.” For these reasons, we chose to include data at ≥30 days.**

3. In line 118, please consider the change of ‘One hundred and thirty’ to ‘130’ as Arabic numbers were used to describe number of patients in line 117 and 119, and 130 should be a large enough number to be used as an Arabic number to start the sentence.

**This has been changed as suggested by the reviewer.**

4. For reference no. 26, it would be consistent to change ‘Accessed 8/2023’ to ‘Accessed August 2023’ as the same style as references no. 1 and 15.

**This has now been changed as suggested by the reviewer.**

5. In table 2, the p-value for univariate analyses in the gender part is 0.0398, which should be considered significant according to the statistical analysis stated. However, the p-value was not bolded. Was this because the significance was adjusted for multiple comparisons? Please explain.

**We have now placed this p-value in bold as suggested by the reviewer.**

6. Figure 2 can be improved by explaining the x-axis and scale in the figure legend.

**We have now made the suggested changes to the figure legend.**

7. "Sex" is considered to be male/female as determined at birth, whereas gender is the male/female by preference and chosen. For this cohort, the use of "sex" seems more applicable. Additionally, line 58 states the PHIS Database uses "sex" as well. Therefore, this seems to be a more applicable term for this study. If indeed this is the case, please change throughout the manuscript.

**We have now made these changes (lines 101 and 151, Tables 1 and 2, and supplemental Table 1) as suggested by the reviewer.**
